# Terminal loop-mediated regulation of miRNA biogenesis: selectivity and mechanisms

**DOI:** 10.1042/BST20130058

**Published:** 2013-07-18

**Authors:** Virginia Castilla-Llorente, Giuseppe Nicastro, Andres Ramos

**Affiliations:** *Cell Biology Program, Sloan-Kettering Institute, Memorial Sloan-Kettering Cancer Center, New York, NY 10065, U.S.A.; †MRC National Institute for Medical Research, The Ridgeway, London NW7 1AA, U.K.

**Keywords:** combinatorial recognition, microRNA biogenesis (miRNA biogenesis), multifunctional protein, protein–RNA interaction, terminal loop, CSD, cold-shock domain, EM, electron microscopy, Exp-5, exportin-5, hnRNPA1, heterogeneous nuclear ribonucleoprotein A1, KSRP, KH (K-homology) splicing regulator protein, Lin28, abnormal cell lineage factor 28, MBNL1, muscleblind-like splicing regulator 1, MCPIP1, MCP-1 (monocyte chemoattractant protein 1)-induced protein, miRNA, microRNA, pre-miRNA, precursor miRNA, pri-miRNA, primary miRNA, RRM, RNA-recognition motif, ssRNA, single-stranded RNA, TDP-43, Tar DNA-binding protein of 43 kDa, TL, terminal loop, TUT4, TUTase4, ZnF, zinc finger

## Abstract

Regulating the expression of individual miRNAs (microRNAs) is important for cell development and function. The up- or down-regulation of the processing of specific miRNA precursors to the mature active form represents one tool to control miRNA concentration and is mediated by proteins that recognize the terminal loop of the RNA precursors. Terminal loop recognition is achieved by the combined action of several RNA-binding domains. The proteins can then regulate the processing by recruiting RNA enzymes, changing the RNA structure and preventing or enhancing the accessibility and processing activity of the core processing complexes. The present review focuses on how terminal loop-binding proteins recognize their RNA targets and mediate their regulatory function(s), and highlights how terminal loop-mediated regulation relates to the broader regulation of mRNA metabolism.

## Introduction

miRNAs (microRNAs) represent a large class of non-coding small RNAs, of ~22 nt in length, predicted to regulate the expression of more than half of the genes encoded in the human genome [1]. They control important developmental processes [2], and deregulation of miRNAs has been implicated in various diseases, including different forms of cancer [3,4]. They base pair imperfectly with the 3′-UTR (untranslated region) of the target mRNA and down-regulate gene expression via translational inhibition and by promoting deadenylation and subsequent degradation of the mRNA [5].

The cellular concentration of miRNAs can be regulated both transcriptionally and post-transcriptionally [6]. Most of the miRNA genes are transcribed by RNA pol (polymerase) II [7]. miRNA genes are located either in non-coding regions or within the intron or exon of protein-coding genes [7]. They can be transcribed either as independent transcriptional units with their own promoters [8] or coincidentally with their host genes. Clusters of several miRNAs can be transcribed as a single long primary transcript [8].

The processing of the pri-miRNA (primary miRNA) transcripts to the mature miRNAs is a multi-step process that is regulated during early development and cellular differentiation, and its misregulation is often associated with human diseases [9]. First, in the nucleus, a ~65-nt hairpin structure, which contains the mature miRNA sequence, called pre-miRNA (precursor miRNA) is cleaved off the pri-miRNA by the Microprocessor complex. The minimal and essential components of this complex are the RNase III enzyme Drosha and its cofactor Dgcr8 (DiGeorge syndrome critical region gene 8). The pre-miRNA is then translocated to the cytoplasm through the nuclear pore complex by the karyopherin Exp-5 (exportin-5). Once in the cytoplasm, another RNase III enzyme named Dicer, in association with TRBP (Tar RNA-binding protein) and PACT [protein activator of PKR (double-stranded-RNA-dependent protein kinase)], recognizes the pre-miRNA hairpin structure and cleaves it to a double-stranded miRNA duplex. This duplex comprises the mature miRNA ‘guidance strand’, which is loaded in the miRISC (miRNA-induced silencing complex), and the quasi-complementary ‘passenger strand’ that is normally degraded [10,11].

The precise processing of pre-miRNA is critical as inaccurate cleavage generates miRNAs with different seed regions, altering the set of genes that a particular miRNA regulates [12]. Regulation of both Drosha and/or Dicer processing has been observed for individual miRNAs [13,14]. However, in most cases, we have only a rudimentary structural insight into the mechanism by which the regulators select their pre-miRNA targets and act upon their processing. The present mini-review focuses on how protein regulators selectively recognize the TLs (terminal loops) of miRNA precursors and on how these protein–RNA interactions are translated into regulatory effects.

## The miRNA precursor TL

The pri-miRNA/pre-miRNA hairpin structures contain mismatches, internal loops and bulges [15]. As for the stem, the TL of these hairpins also has a variable structure, often comprising ssRNA (single-stranded RNA) regions. The TL structure may be important to influence the rate at which a specific miRNA is produced, and structural and functional tools have helped to explore the role played by the TL in regulating the different processing steps.

It was proposed that a flexible and long (≥10 nt) TL is important for processing by Drosha because reducing the length or changing the sequence of a TL affected pri-miRNA processing efficiency significantly [16]. However, Han et al. [17] performed a detailed mutational analysis of pri-*miR-16-1* processing and concluded that the conformation of the TL is generally not important for the processing, although a degree of flexibility in the TL may be beneficial.

The nuclear export of the pre-miRNA is unlikely to be influenced directly by subtle changes in the structure of the TL. In the high-resolution structure of the pre-miRNA nuclear export machinery [18], the pre-miRNA is packed in a baseball mitt-like structure formed by the Exp-5–RanGTP complex. The structure showed that the 2-nt 3′ overhang and the mostly double-stranded RNA stem region are the pre-miRNA features key to the interaction with Exp-5, rather than the TL. The structure also showed that protein recognition relies on the RNA helical structure rather than on its sequence.

In contrast, in the cytoplasm, the Dicer protein could contact the pre-miRNA TL. A recent three-dimensional reconstruction of the large human Dicer with EM (electron microscopy) used wild-type and mutated protein constructs to fit high-resolution structures of single domains in a lower-resolution EM map [19]. The reconstruction showed an L-shaped molecule, with the three helicase domains forming the base of the L and potentially making contact with the TL. Instead, deletion of the helicase domains has little effect on Dicer–pre-miRNA binding affinity, although it results in faster pre-miRNA processing kinetics [20].

In general, the TL seems to be largely dispensable for the core activity of Drosha, Dicer and Exp-5, consistent with the variability in its structure, length and sequence in different miRNAs. However, the TL has been shown to be important for the processing of specific miRNAs or groups of miRNAs and, in some cases, a regulatory mechanism that involves *trans*-acting protein factors binding to the TL has been identified [21,22]. Furthermore, a significant number of miRNA precursors has highly conserved TLs and it seems likely that, for many of these precursors, the TL represents a *cis*-regulatory element that acts as a binding platform for proteins that up- or down-regulate miRNA biogenesis [23].

## Recognition of the pre-miRNA TL by multifunctional RNA-binding proteins

**Figure 1:**
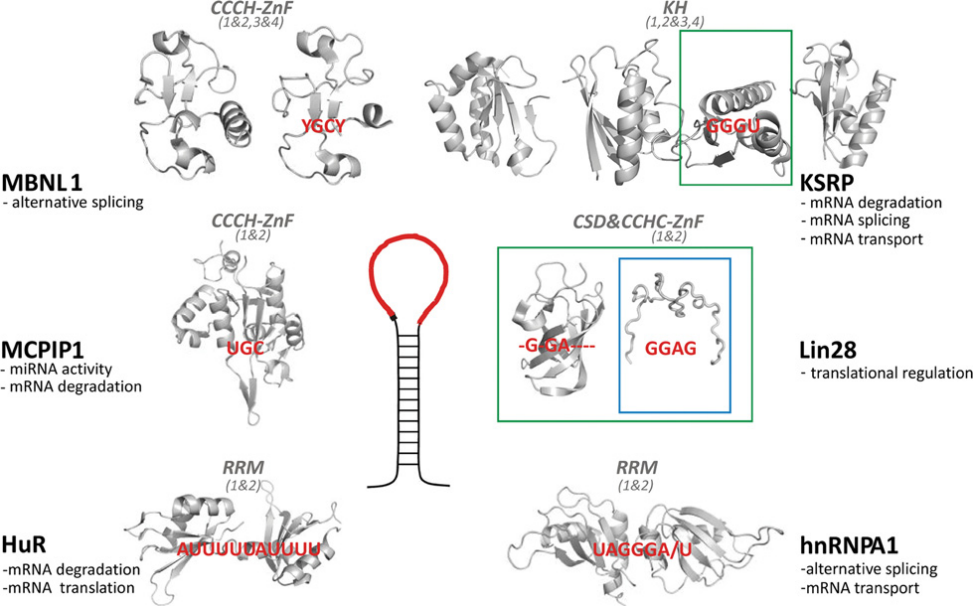
Proteins recognizing the miRNA precursor TL and regulating miRNA biogenesis have multiple roles in RNA metabolism Additional functions of the proteins are below the protein's name. TL/RNA-target recognition is mediated by several domains, each interacting with a short RNA sequence. Cartoon representations of the RNA-binding domains in grey (PDB codes: HuR, 4EGL; MCPIP1, 3V33; MBNL1, 3D2N and 3D2Q; KSRP, 2OPU, 2JVZ and 2HH2; Lin28, 3ULJ and 2CQF; hnRNPA1, 1UP1) and the recognized sequences in red. Domain type and domain numbering within the protein are in italic, domains that bind RNA as di-domain structures are grouped using ‘&’ symbols. For Lin28 and KSRP, structures of both isolated domains and larger construct are available in complex with RNA targets. The domains for which such structures are available are colour-boxed [26,28,33].

The proteins described to regulate Drosha and/or Dicer processing by interacting with the TL of specific miRNA precursors possess known RNA-binding domains and perform additional functions in RNA metabolism (Figure 1).

Lin28 (abnormal cell lineage factor 28) interacts with the miRNA precursors of the *let-7* family, regulating their processing and turnover [24]. Lin28 contains two RNA-binding domains, an N-terminal CSD (cold-shock domain) and a C-terminal double CCHC-type (Cys-Cys-His-Cys) ZnF (zinc finger) domain. The CSD and ZnF domains recognize different elements of the TLs of the miRNA precursors. Structural and biophysical data on the interaction between Lin28 and a number of *let-7* precursors confirm that the main role of the CSD domain is to increase the affinity of Lin28 for the target, although some sequence selectivity exists [25–27] (Figure 1). The double-CCHC ZnF domain recognizes a specific GGAG sequence in the pre-*let-7* TL [25,26,28] and is thought to provide most of the specificity of the Lin28–RNA interaction. Finally, the crystal structure of Lin28 in complex with three different *let-7* precursors has provided a model to explain how the two RNA-binding domains of Lin28 together may accommodate different RNA secondary structures [26]. Interestingly, two recent studies have examined the distribution of Lin28 on the cellular transcriptome and have highlighted the importance of the protein in up-regulating translation of specific mRNAs [29,30].

KSRP [KH (K-homology) splicing regulator protein] is a multifunctional protein that interacts with its nucleic acid targets using four consecutive KH domains [31,32]. The four domains act combinatorially, although their contribution varies depending on the target [31–34]. This plasticity allows the recognition of a broad range of targets and the engagement of KSRP in different steps of RNA regulation, including mRNA splicing, transport and decay, as well as miRNA biogenesis. In the latter, KSRP recognizes the loop of the precursors of several *let-7* family members, and of a small number of other miRNA precursors [35,36]. Structural and biophysical data have described how KH3 plays a dominant role in KSRP interaction with pre-*let-7a*, recognizing a G-rich site in the TL [33,35].

hnRNPA1 (heterogeneous nuclear ribonucleoprotein A1) is another RNA-binding protein implicated in miRNA biogenesis. The two RRMs (RNA-recognition motifs) of hnRNPA1 form a structural unit that recognizes a G-rich sequence, mediating the function of the protein as an alternative splicing factor as well as a positive (for pri-*miR18a*) and negative (for pri-*let-7a*) regulator of pri-miRNA processing [23,37–39]. TDP-43 (Tar DNA-binding protein of 43 kDa), like hnRNPA1, contains a tandem RRM domain which binds preferentially to GU-rich sequences acting as a splicing regulator [40]. TDP-43 also interacts with the TL of the precursors of *miR-574* and *miR-143* promoting Drosha and Dicer processing [41]. Similarly, the splicing regulator FUS interacts with the TL of a group of miRNAs with a role in neuronal function promoting Drosha recruitment and processing [42]. Like hnRNPA1, FUS contains an RGG-box as well as a RRM domain, but has lower sequence specificity than hnRNPA1 or TDP-43. Finally, HuR is a multifunctional regulator of RNA stability and recognizes a set of AU-rich sequences using its two N-terminal RRM domains [43]. It has been shown recently that the recognition of *miR-7* TL by HuR inhibits pri-miRNA processing [44].

In summary, the regulators of RNA biogenesis are multifunctional proteins not only involved in miRNA biogenesis, but also playing a more global role in tuning RNA metabolism. They contain multiple domains, each recognizing short ssRNA sequences, and use them in a co-ordinated fashion to select the RNA targets (Figure 1). The proteins’ temporal expression and cellular localization, as well as the key RNA-recognition features, are likely to play a role in defining which of the functionally and structurally diverse RNA targets are bound by the protein at a given time and in a given cell type.

Only a small number of regulatory protein–TL interactions have been characterized to date, but, on the basis of the number of miRNAs with conserved TL, it seems likely that many more RNA-binding proteins will be identified as active regulators of miRNA biogenesis. A recent approach to test the binding of proteins to miRNA in cellular extract and in the cell [45] could be a predictor for potential effects on the biogenesis of specific miRNAs *in vivo*. Exploring the interaction under different conditions and in different cell types would expand this understanding.

## Regulatory mechanisms

The mechanisms used by the regulatory proteins to alter miRNA biogenesis include TL cleavage, the recruitment or displacement of other proteins with catalytic activity, the sequestering of the miRNA precursor away from the processing machineries, and the induction of changes in the RNA structure and dynamics.

Lin28 is expressed from two different foci resulting in two proteins, Lin28a and Lin28b. Both have been implicated in *let-7* miRNA processing, but at different steps of the processing pathway. Lin28b is mostly nuclear, with a specific localization in the nucleolus. Upon interaction with Lin28b, the *let-7* pri-miRNA is sequestered in the nucleolus, which inhibits pri-*let-7* processing by Drosha [46]. The mechanism of action of Lin28a is different. The protein interacts with the TL of pre-*let-7* in the cytoplasm, inducing changes in the RNA secondary structure at the Dicer cleavage site, inhibiting Dicer processing [26,47]. Lin28a also recruits the non-canonical poly(A) polymerase TUT4 (TUTase4) [25] that uridylates the 3′ end of the precursor directing the pre-miRNA to degradation [48]. Moreover, it has been shown that the Lin28–TUT4 mechanism can also regulate the processing of *miR-1*, and this activity can be blocked by the alternative splicing factor MBNL1 (muscleblind-like splicing regulator 1). This protein can out-compete Lin28, acting as a positive regulator of *miR-1* biogenesis [49].

In the case of MCPIP1 [MCP-1 (monocyte chemoattractant protein 1)-induced protein], the molecular mechanism affecting miRNA biogenesis is totally different. This protein is a ribonuclease that interacts and cleaves the TL of pre-miRNAs counteracting Dicer processing. This leads to a down-regulation of mature miRNA production. It has been proposed that binding of proteins to the pre-miRNA TL in the cytoplasm could antagonize the effects of MCPIP1 by protecting the precursor from the cleavage [50].

**Figure 2:**
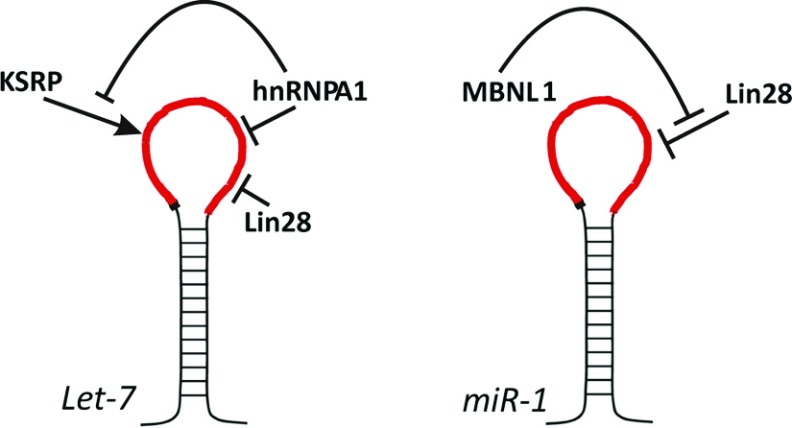
Several protein regulators can interact with the same TL creating a complex regulatory network The regulatory network of *let-7* and *miR-1* exemplifies this concept.

KSRP up-regulates both Drosha and Dicer processing. It contacts both proteins, and could act by optimizing their recruitment and/or positioning in the processing complexes [35]. *In vitro*, KSRP also modulates the structure of the *let-7* TL, freezing it in one of two possible conformations (G. Nicastro, D. Hollingworth and A. Ramos, unpublished work). Interestingly, this conformation is alternative to the one that is imposed by Lin28 binding, which distorts the Dicer cleavage site. In the case of hnRNPA1, regulation of *miR-18* relies on the induction of changes in the structure or dynamics of the RNA. hnRNPA1 binds to the pri-*miR-18* stem and TL, inducing a relaxation of the secondary structure of the precursor miRNA which creates a more favourable cleavage site for Drosha [23,38]. Interestingly, hnRNPA1 binding to pri-*let-7a* has the opposite effect to that observed on *miR-18*, reducing its processing efficiency. It has been proposed this effect derives, at least in part, from the competition between hnRNPA1 and KSRP for their common G-rich target sequence on pre-*let-7* TL [39]. Targeting of the same TL by different protein regulators seems to be common in TL-mediated regulation of miRNA biogenesis (Figure 2).

Interestingly, in a number of cases, the regulation of pri- and pre-miRNA processing is mediated by structural changes in miRNAs precursors that arise from chaperone-like activities of RNA-binding proteins. However, the current level of structural understanding of the processing complexes and of the miRNA precursors is still sketchy, which hinders a detailed examination of these mechanisms.

## Conclusion

TL-mediated regulation of miRNA biogenesis regulates processes ranging from cellular proliferation to inflammation and muscle and neuronal development. The study of this regulation is in its infancy, but some trends and challenges have started to emerge.

The selectivity of TL-mediated regulation relies on the recognition of the TL of a particular RNA, which is achieved via the specific recognition of RNA nucleobases in ssRNA regions by multiple RNA-binding domains. In addition, the structure of the loop is also likely to be important by regulating the accessibility of the single-stranded sequences to the protein domains and directly mediating the regulatory effect. High-resolution information on the structure and dynamics of the protein regulators and of the Microprocessor and Dicer machineries, in complex with the miRNA precursors, is necessary to understand how the hairpin structure mediates the effect of protein binding on miRNA biogenesis.

Only a few examples of TL-mediated regulation have been dissected to date. The combinatorial RNA recognition properties of the protein regulators, their diverse mechanism(s) of action and the convergence of several pathways on the same TLs point towards the existence of a complex regulatory network of proteins. Most of the protein regulators have additional function in mRNA metabolism, which highlights the links and similarities between miRNA regulatory networks and the regulation of the different steps of RNA metabolism.

In the next few years, the screening of protein–RNA interactions and the regulatory functions of RNA-binding proteins are likely to show that many RNA-binding proteins playing a role in mRNA regulation are also active in the TL-mediated regulation of specific miRNAs, which will expand the repertoire of known regulatory mechanisms. The molecular characterization of these mechanisms will hopefully provide tools to selectively interfere with the concentration of miRNAs important to human health and disease.
